# Hamstring Stiffness and Strength Responses to Repeated Sprints in Healthy Nonathletes and Soccer Players With Versus Without Previous Injury

**DOI:** 10.1177/19417381231175474

**Published:** 2023-05-31

**Authors:** Sandro R. Freitas, Régis Radaelli, Raúl Oliveira, João R. Vaz

**Affiliations:** † Laboratório de Função Neuromuscular, Faculdade de Motricidade Humana, Universidade de Lisboa, Cruz-Quebrada, Lisboa, Portugal; ‡ Egas Moniz Center for Interdisciplinary Research, Egas Moniz School of Health and Science, Monte de Caparica, Setúbal, Portugal; § CIPER, Faculdade de Motricidade Humana, Universidade de Lisboa, Cruz-Quebrada, Lisboa, Portugal

**Keywords:** biceps femoris long head, hamstring injury, rate of force development, semitendinosus, shear wave elastography, soccer

## Abstract

**Background:**

The effect of 10 × 30 m repeated sprints on passive and active stiffness of semitendinosus (ST) and biceps femoris long head (BFlh), and knee flexor maximal voluntary isometric contraction (MVIC) and rate of force development (RFD), and whether athletes with previous hamstring injury have a different response, is unknown.

**Hypothesis:**

Repeated sprints would (1) increase BFlh stiffness and decrease ST stiffness and knee flexors MVIC and RFD in healthy participants; and (2) greater magnitude of response would be seen in athletes with previous hamstring injury.

**Study Design:**

Case series (experiment I) and case control (experiment II) study designs.

**Level of Evidence:**

Level 3.

**Methods:**

Healthy nonathletes attended 2 replicated sessions (experiment I, n = 18), while soccer players with (n = 38) and without (n = 67) previous hamstring injury attended 1 testing session (experiment II).

**Results:**

In both experiments, the knee flexors MVIC and RFD decreased after the sprints (*P* < 0.05). In experiment I, the ST and BFlh passive stiffness reduced after the sprints (*P* < 0.02), while a small BFlh active stiffness increase was noted (*P* = 0.02); however, no correlation was observed between the 2 testing sessions for the postsprint muscle stiffness responses (*r* = -0.07-0.44; *P* > 0.07). In experiment II, only an ST passive stiffness reduction was observed after the sprints (*P* < 0.01). No differences were noted between injured and noninjured lower limbs for any variable (*P* > 0.10).

**Conclusion:**

Repeated sprints are likely to decrease the knee flexor’s maximal and rapid strength, and to alter the hamstring stiffness in the nonathlete population. Previous hamstring injury does not apparently affect the footballer’s hamstring functional and mechanical responses to repeated sprints.

**Clinical Relevance:**

The responses of hamstring stiffness and knee flexor strength to repeated sprints are unlikely to be associated with hamstring injury.

The incidence of hamstring injuries in team athletes that involve repeated running sprints is commonly reported as high,^9,11^ but only the hamstring injury history and age have been consistently identified as risk factors.^19^ This type of injury affects mainly the biceps femoris long head (BFlh) and, to a lesser extent, the semitendinosus (ST),^13^ and occurs mostly during actions that require rapid muscle activation such as sprinting.^9,11^ However, inconsistent findings have been reported concerning the sprinting performance and knee flexors strength capacity of athletes with versus without previous hamstring injury.^5,2,29,30,31^ For instance, some studies have reported that previously injured athletes are faster,^22^ more susceptible to fatigue during the repeated sprints,^31^ but with lower knee flexors rate of force development (RFD)^5,18,30^; other studies have obtained different conclusions.^26,29^ Although previous investigations have shown that performing repeated sprints reduces sprint ability,^2,12^ these investigations have not examined the effects on knee flexor strength. In particular, the RFD of the knee flexors is an area of interest given the suggestions of relevance to the prevention of hamstring injuries.^5,30^ Thus, this topic needs to be further explored.

The stiffness of hamstring muscles, whether passive or active,^15,27^ has also been suggested by some studies as potentially relevant for understanding hamstring injuries. However, surprisingly, little has been investigated at this level, including the response of muscle stiffness after performing fatigue-inducing tasks such as repeated sprints. Interestingly, based on functional MRI measurements, it was previously proposed that an altered load distribution between hamstring muscles, in particular between BFlh and ST, could underlie the occurrence of hamstring injuries.^32,33^ Considering that active muscle stiffness represents in a way the load that the muscle develops in contraction,^21^ and based on the previous premise,^32,33^ it was recently observed that the responses of active stiffness of the BFlh and ST are distinct during submaximal knee flexors contractions (ie, ST > BFlh),^25^ change over the course of fatigue (ie, ST decreases and BFlh is unaltered),^24^ and are apparently different in elite soccer players with a history of hamstring injury.^15^ Specifically, soccer players with previous hamstring injury appear to have less active BFlh stiffness in a nonfatigue state, and tend to develop a greater increase in BFlh/ST active stiffness ratio during the course of fatigue onset.^15^ However, the active stiffness response of these hamstring muscles over the fatigue after repeated sprints has not yet been investigated, and could potentially provide relevant information.

The present study aimed to further explore the immediate effects of repeated sprinting on hamstring stiffness and knee flexors strength capacity in both healthy nonathletes and soccer players with and without previous hamstring injury in the last 2 years. Thus, 2 experiments were conducted. The first experiment focus was 2-fold: (1) to examine whether BFlh and ST passive and active stiffness, as well as the knee flexors maximal voluntary isometric contraction (MVIC) and RFD, would be altered by a repeated sprinting task in healthy participants; and (2) to determine whether muscle stiffness responses to the repeated sprinting task between the 2 replicated sessions would be associated. It was hypothesized that after the sprint task the knee flexor MVIC and RFD would reduce^26,29^; and that under the absence of changes in muscles passive stiffness, ST active stiffness would be reduced and BFlh active stiffness would be increased.^15^ In (2) muscle stiffness responses would be similar between sessions.^24^ The second experiment examined whether soccer players with and without previous hamstring injury would present differences in repeated sprints performance, and verified whether previously injured and noninjured lower limbs would present differences in ST and BFlh stiffness and knee flexors MVIC and RFD before and after repeated sprints. It was hypothesized that previously injured athletes would present similar sprint performance, and previously injured limbs would have a greater active stiffness response in terms of BFlh increase and ST decrease after the sprints in the absence of changes in muscle passive stiffness,^15^ but with similar knee flexor MVIC and RFD responses.^26,29^

## Methods

### Study Design and Sample

Descriptive (experiment I) and a controlled (experiment II) laboratory study designs were implemented. Experiment I replicated the testing session with 7.3 ± 2.0 days between sessions, whereas experiment II consisted of only 1 session performed at the 2021/2022 preseason (ie, July-August 2021). The experiments had a similar protocol, consisting of muscle stiffness and knee flexors strength pretesting, standardized warm-up, sprint testing, and post testing (described in the “Protocol” section). For experiment I, participants were instructed to avoid the practice of vigorous physical activity but to maintain normal daily living, whereas in experiment II soccer players were coming from a vacation period, or from the beginning of the season. Based on preliminary statistical testing using G*Power software (Version 3.1.9.3, Universität Düsseldorf), 18 and 100 participants were estimated as necessary for experiments I and II, respectively, assuming statistical power of 90% in the main outcomes (ie, muscle stiffness and knee flexors strength) and that at least 20% of footballers would present with previous hamstring injury.^13^ Healthy and physically active male adults (n = 18; 25.2 ± 4.8 years, 79.1 ± 10.8 kg, 1.78 ± 0.06 m) participated in experiment I, and none reported having previous hamstring injury. Semiprofessional (n = 60) and professional (n = 45) soccer players (excluding goalkeepers; 24.8 ± 4.2 years, 73.6 ± 7.6 kg, 1.78 ± 0.06 m) from 10 Portuguese teams competing in the first (n = 2), second (n = 19), third (n = 24), and fourth (n = 60) divisions agreed to participate in experiment II. At the time of testing, athletes reported to be free of clinical injuries. This study was approved by the local ethics committee (approval number: 9/2019).

### Equipment and Variables

#### Knee Flexor Strength

Knee flexor torque was measured with a sampling rate of 1 kHz using a piece of custom-built equipment as shown in a previous study.^24^ Participants were positioned in a prone position, with the hips in neutral position, and the tested knee flexed at 30º (0º = full extension). With the ankle at 90º, the foot of the tested limb was fixed in a foot holder, which contained a force transducer (Model STC, Vishay Precision) near heel level to collect the linear force perpendicular to leg orientation. The force collected was amplified (Model UA73.202, Sensor Techniques), converted digitally (USB-230 Series, Measurement Computing Corp), recorded using DAQami software (Version 4.1, Measurement Computing Corp), and multiplied by the perpendicular distance between the force transducer center and the femoral lateral condyle to estimate knee torque.

Knee flexor MVIC and RFD were obtained during unilateral testing in experiment I, and bilateral testing in experiment II. In a previous pilot testing (not published) we observed no differences in knee flexor MVIC and RFD between unilateral and bilateral testing. For each testing moment (ie, before and after the sprint task), athletes performed 2 trials with 60 seconds between trials. In experiment II, testing was randomized between limbs. For each trial, participants were instructed to relax as much as possible, and after the command “3, 2, 1, and go” to contract the knee flexors “as fast and strong” as possible, without performing a countermovement. Visual feedback was provided to the athletes. When visually verified that countermovement had occurred, an additional trial was performed.

#### Muscle Stiffness

BFlh and ST passive and active stiffness were assessed using 2 similar ultrasound scanners (ie, Aixplorer [Version 11 software] and Aixplorer Ultimate [Version 13 software]; Supersonic Imagine) in shear wave elastography mode (musculoskeletal preset, penetrate mode, smoothing level 5, persistence off; scale: 0-100 kPa for passive measurements, and 0-800 kPa for active measurements). Each scanner was coupled with a linear transducer array (SL10-2, 2-10MHz, Vermon). The push frequency that generated the elastogram window was set automatically by the ultrasound equipment to approximately 1 Hz (range, 0.8-1.4 Hz), depending on the elastogram window size and position. The region of interest was defined at ~45% of the proximal-to-distal femur length for both muscles,^25^ with the use of plastic casts fixed to the skin using double-sided adhesive tape, to ensure the measurement site was the same across repeated trials. Casts were oriented according to the fascicle direction of the muscle. Measurements were conducted by examiners trained by the principal investigator of this study, with a minimal ultrasound training time of 50 hours. Minimum pressures were applied by examiners during the stiffness measurements. Active stiffness measurements were obtained during a knee flexor isometric contraction at 20% of MVIC. This contraction intensity was chosen since it has been proven to be reliable, is acceptable to athletes, because ST displays greater active stiffness than BFlh,^25^ and previous research has indicated the injured limbs of footballers have different responses compared with controls.^15^ Videos were recorded with both B-mode and elastogram windows, with a minimum of 30 seconds for both passive and active measurements, with 30 seconds rest between trials. For further details of data acquisition and processing, please refer to earlier works.^24,25^

#### Sprint Velocity

Using a previous published approach,^6^ the time to perform the 10 sprints of 30 m each was assessed using wired timing gates provided by Chronojump-Boscosystem (Velleman PEM10D photocell), connected to the Chronojump software (Version 2.1.1-16, Chronojump Boscosystem). Photocells were placed at a distance of 30 m, at a height of 1.3 m. Sprints were performed on an indoor tartan floor, whereas participants positioned 1 m before each pair of photocells, and initiated each sprint upon a “3, 2, 1, and go” command by a local instructor. Participants rested 30 seconds between sprints.

### Protocol

Demographic, anthropometric, and injury history information was obtained upon arrival. With the participant lying in a prone position and the knees flexed at ~30°, the ST and BFlh region of interest were determined, and the muscle passive stiffness assessment performed. After a brief familiarization with the knee flexors strength equipment, athletes performed the MVIC/RFD testing followed by the ST and BFlh active stiffness assessment (in random order). Before the sprinting task, participants performed a standardized warm-up (ie, 5 minutes of low intensity running, followed by 8 minutes of dynamic mobilization, and calisthenics of the lower limb). Immediately after the sprints, muscle active stiffness, knee flexor MVIC and RFD, and the muscle passive stiffness were assessed. In experiment I, only 1 lower limb (randomized between athletes) was tested for muscle stiffness and for knee flexor strength, whereas in experiment II the 2 lower limbs were assessed in random order.

### Data Processing

Knee flexor MVIC and RFD were processed using a customized Matlab routine (Mathworks Inc). For MVIC, the highest value observed in pre- and postsprints trials was considered for analysis. For RFD, the torque slope at the 0 to 50, 50 to 100, 100 to 150, and 150 to 200 ms time intervals after the onset of muscle contraction (ie, defined as 3 SDs from the baseline), as well as the maximum RFD (ie, maximal tangential slope between 2 adjacent datapoints observed in the first 200 ms) and its moment of occurrence after the onset of muscle contraction were determined.

Shear modulus data were processed using custom Matlab routines (Mathworks Inc). Briefly, each clip exported from Aixplorer software was sequenced into .jpeg images. The largest rectangular region of interest in the elastogram window in each image was selected manually by avoiding aponeuroses and tissue artifacts (eg, vessels). Grayscale pixels were excluded, and colored pixels were converted into elastic moduli values based on the recorded scale. The elastic moduli values were then averaged to obtain a representative muscle value. In each knee flexor isometric contraction trial, the average value observed during the most stable period of 15 seconds was determined,^25^ and the average between the 2 repeated trials of each testing moment was considered for analysis.

Concerning the repeated sprint performance, in addition to the sprint velocity in each repetition, the percentage of drop in velocity between the first and the last sprint repetitions was considered for analysis.

### Statistical Procedures

SPSS software (Version 26, IBM Corporation) was used to conduct statistical analysis. Data normal distribution was checked using Shapiro-Wilk test.

In experiment I, a 2-way repeated measures analysis of variance (ANOVA) (session [1, 2] × repetition [1, 2, 3, 4, 5, 6, 7, 8, 9, 10]) with simple contrast against the first sprint repetition was performed to examine whether the velocity decreased along the repeated sprint task in both sessions, whereas the maximal sprint velocity and the percentage drop of velocity during the sprint task between sessions were compared using a paired *t* test. The effects of sprinting in both sessions on the BFlh and ST passive and active stiffness and knee flexor MVIC and RFD were examined using a 2-way repeated measures ANOVA [session [1, 2] × moment [pre, post]). Based on pretesting values, interday reliability outcomes (ie, intraclass correlation coefficient, and typical error) were also quantified. Pearson correlation coefficient was used to examine whether the active stiffness responses of BFlh and ST after the sprint task were consistent between sessions.

In experiment II, an unpaired *t* test was performed to compare age, height, body mass, and body mass index between athletes with versus without previous injury. To examine whether the repeated sprint task performance were different between previously injured and noninjured athletes, a 2-way repeated measures ANOVA (group [injured, noninjured) × repetition [1, 2, 3, 4, 5, 6, 7, 8, 9, 10]) with simple contrast against the first sprint repetition was conducted, and a unpaired *t* test was used to compare the maximal sprint velocity and the percentage drop of velocity during the sprint task between groups. To compare the effects of sprinting on the BFlh and ST passive and active stiffness and the knee flexor MVIC and RFD between previously injured and noninjured lower limbs, a 2-way repeated measures ANOVA [limb [injured, noninjured] × moment [pre, post]) was conducted.

In all ANOVAs, post hoc testing consisted of the Bonferroni test. Statistical significance was set at *P* > 0.05.

## Results

### Experiment I

**Figure 1.**
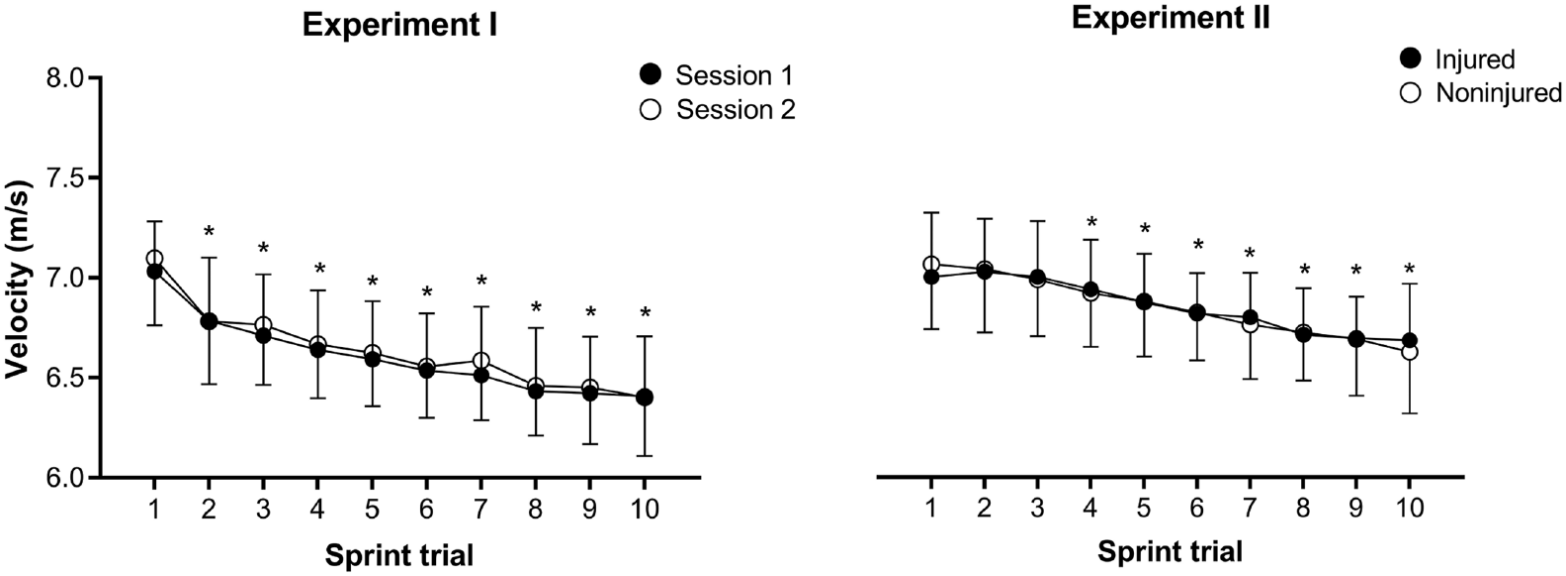
Sprint velocity observed in the repeated sprint task performed by healthy participants in 2 replicated sessions (experiment I, left panel) and by soccer players with versus without previous hamstring injury (experiment II, right panel). *Statistically different from the first sprint trial (*P* < 0.05).

In the repeated sprint task, the sprint velocity decreased from the second repetition in both testing sessions (Figure 1, left panel), whereas an effect was found for the repetition factor (*P* < 0.01), but not for the session factor (*P* = 0.24) nor the session × repetition interaction (*P* = 0.86). Participants showed similar maximal sprint velocity (session 1, 7.0 ± 0.2 m/s; session 2, 7.1 ± 0.3 m/s; *P* = 0.21) and percentage drop of velocity (session 1, 8.9 ± 3.2%; session 2, 9.7 ± 3.7%; *P* = 0.28). Thus, suggesting that participants had similar sprint performance between the 2 testing sessions.

Table 1 summarizes the descriptive data (ie, means and standard deviations) and ANOVA outcomes of BFlh and ST passive and active stiffness, knee flexors MVIC and RFD, and the time when the maximum RFD occurred, as well as the intersession reliability outcomes for the pretesting values. No effect was noted for the session factor, as well as for the session × moment interaction, for any variable. In contrast, an effect was noted (*P* < 0.05) for BFlh active stiffness, RFD (at all time intervals), and time when the maximum RFD occurred.

**Table 1.** Hamstring (active and passive) stiffness and knee flexors maximal and rapid strength before and after a repeated sprints task in 2 replicated sessions, as well as their ANOVA and intersession reliability outcomes^*a*^

Variables		Descriptive Values				ANOVA Outcomes						Reliability Outcomes^*^a^*^		
		Session 1		Session 2		Session × factor		Moment × factor		Session × Moment		Intersession		
		Pre	Post	Pre	Post	*P*	η_p_^2^	*P*	η_p_^2^	*P*	η_p_^2^	ICC [95% CI]	SEM	*P*
Active stiffness, kPa	BFlh	25.0±8.6	27.7±9.5	23.9±7.6	25.7±9.5	0.24	0.079	0.024	0.265	0.40	0.043	0.69 [0.35-0.87]	4.6	0.49
	ST	56.7±20.7	54.1±16.3	58.0±19.0	53.4±19.0	0.93	<0.001	0.123	0.134	0.48	0.029	0.67 [0.30-0.86]	11.6	0.75
Passive stiffness, kPa	BFlh	4.0±0.5	3.7±0.6	4.3±1.2	4.0±1.6	0.41	0.042	0.012	0.318	0.93	<0.001	-0.01 [-0.46-0.46]	0.9	0.37
	ST	4.4±0.8	3.8±0.8	4.4±0.9	4.2±0.9	0.19	0.10	0.020	0.281	0.20	0.097	0.83 [0.60-0.93]	0.3	0.82
MVIC, Nm		117.1±20.0	108.2±19.1	118.3±18.7	106.5±22.1	0.92	0.01	0.002	0.441	0.49	0.029	0.76 [0.47-0.90]	9.6	0.71
RFD, Nm/ms	0-50 ms	0.41±0.11	0.30±0.10	0.39±0.10	0.33±0.10	0.87	0.01	<0.001	0.741	0.11	0.147	0.42 [-0.02-0.74]	0.08	0.31
	50-100 ms	0.67±0.12	0.53±0.11	0.68±0.14	0.56±0.15	0.36	0.05	<0.001	0.685	0.34	0.054	0.59 [0.18-0.83]	0.08	0.82
	100-150 ms	0.51±0.09	0.47±0.09	0.54±0.08	0.47±0.10	0.22	0.09	0.002	0.445	0.16	0.114	0.63 [0.25-0.84]	0.05	0.09
	150-200 ms	0.33±0.09	0.33±0.08	0.35±0.08	0.31±0.07	0.76	0.01	0.215	0.089	0.15	0.116	0.68 [0.35-0.87]	0.05	0.19
	max RFD, ms	0.74±0.16	0.58±0.13	0.74±0.14	0.61±0.16	0.49	0.03	<0.001	0.691	0.56	0.020	0.63 [0.24-0.85]	0.09	0.83
Time RFD max, ms		68.2±15.0	82.2±21.8	70.8±13.2	73.7±14.1	0.40	0.04	0.046	0.214	0.14	0.126	0.05 [-0.44-0.50]	13.8	0.58

BFlh, biceps femoris long head; CI, confidence interval; ICC, intraclass correlation coefficient; MVIC, maximal voluntary isometric contraction; max RFD, maximal rate of force development; η_p_^2^, partial eta square; *P*, *P* value; RFD, rate of force development; SEM, standard error of measurement; ST, semitendinosus; Time RFD max, time when occurred the maximal rate of force development.

a Reliability outcomes were determined for the pretesting data of the 2 replicated sessions by calculating the ICC, the SEM, and a paired *t* test.

No relation was found in BFlh (active, *r* = 0.12, *P* = 0.64; passive, *r* = 0.26, *P* = 0.30) and ST (active, *r* = -0.07, *P* = 0.78; passive, *r* = 0.44, *P* = 0.07) stiffness responses between testing sessions, suggesting that stiffness responses to repeated sprinting are inconsistent between days.

### Experiment II

Due to data recording errors, data from 1 athlete in the sprint testing, 3 athletes from the passive stiffness testing, and 1 athlete from the active stiffness testing were not considered for analysis.

Table 2 summarizes the characteristics of 105 athletes with and without previous hamstring injury that participated in study. Among the soccer players who completed the testing, 38 athletes (36.2%) reported having ≥1 previous hamstring injury in the last 2 years resulting from football practice. In only 59.5% (n = 25) of the injuries were athletes able to identify the injured muscles, whereas 64.0% (n = 16) reported the biceps femoris to be affected. Injuries were reported to occur during sprinting (50%), stretching actions (11.9%), and other situations (38.1%).

**Table 2.** Profile of the 105 footballers with and without previous hamstring injury who participated in experiment II

	Footballers			
	Previous Injury	No Previous Injury	*P* Value^*a*^	Effect Size
n, total	38	67	-	-
Age, y	26.0±4.2	24.0±4.1	0.02	0.48
Height, m	1.78±0.06	1.78±0.06	0.50	0.0
Body mass, kg	74.7±8.2	73.1±7.2	0.30	0.39
Body mass index, kg/m^2^	23.6±1.5	22.9±1.5	0.02	0.47
Central defenders, n	7	16	-	-
Lateral defenders, n	11	11	-	-
Central midfielders, n	11	20	-	-
Side midfielders, n	4	11	-	-
Forwards, n	5	9	-	-
Right limb dominance, %	73.7	71.6	-	-
Total hamstring injuries, n	42	-	-	-
Unilateral hamstring injuries, n	34	-	-	-
Dominant limb hamstring injuries, n	30	-	-	-
Injury context: game; training; and other, n	17; 24; 1	-	-	-
Injury time loss, days	25.4 ± 17.2	-	-	-
Time from injury, mo	13.5 ± 6.9	-	-	-

a *P* value refers to an unpaired *t* test conducted to compare between footballers with versus without previous hamstring injury, and the effect size to the Cohen’s *d* effect size.

Regarding the repeated sprint performance, no effect was observed for the group factor (*P* = 0.94) nor the group × repetition interaction (*P* = 0.26), but an effect was noted for the repetition factor (*P* < 0.01; Figure 1, right panel), whereas sprint velocity decreased from the fourth repetition (*P* < 0.01). In addition, similar maximal sprint velocity (injured, 7.0 ± 0.3 m/s; noninjured, 7.1 ± 0.3 m/s; *P* = 0.34) and percentage drop of velocity (injured, 4.5 ± 5.3%; noninjured, 6.2 ± 5.5%; *P* = 0.12) was observed between groups.

Table 3 presents the values (means and standard deviations) of knee flexor strength and muscle stiffness outcomes for lower limbs with and without previous hamstring injury, as well as their ANOVA outcomes. The ST passive stiffness and knee flexor MVIC and RFD (in all intervals) decreased after the sprint task, but no differences were observed between limbs with versus without previous hamstring injury for any variable.

**Table 3.** Hamstring (active and passive) stiffness and knee flexors maximal and rapid strength before and after a repeated sprints task in footballers with and without previous hamstring injury, as well as their ANOVA outcomes

Variables		Limbs				ANOVA Outcomes					
		Previous Injury		No Previous Injury		Group Factor		Moment Factor		Group × Moment	
		Pre	Post	Pre	Post	*P*	η_p_^2^	*P*	η_p_^2^	*P*	η_p_^2^
Active stiffness, kPa	BFlh	29.6 ± 8.9	29.9 ± 11.6	32.2 ± 10.0	32.4 ± 11.4	0.13	0.011	0.76	<0.001	0.99	<0.01
	ST	59.4 ± 20.5	56.1 ± 15.6	58.7 ± 15.3	59.7 ± 15.2	0.59	0.001	0.23	0.007	0.03	0.02
Passive stiffness, kPa	BFlh	4.3 ± 0.6	4.2 ± 0.8	4.5 ± 0.8	4.4 ± 0.9	0.14	0.011	0.32	0.005	0.95	<0.01
	ST	4.5 ± 0.6	4.1 ± 0.7	4.7 ± 0.7	4.3 ± 0.7	0.10	0.013	<0.01	0.208	0.44	0.01
MVIC, Nm		142.9±37.7	132.4±35.8	139.6±26.0	128.6±25.3	0.46	0.003	<0.01	0.307	0.86	<0.01
RFD, Nm/ms	0-50 ms	0.48 ± 0.19	0.44 ± 0.18	0.44 ± 0.19	0.41 ± 0.16	0.20	0.008	0.02	0.024	0.79	<0.01
	50-100 ms	0.85 ± 0.27	0.75 ± 0.22	0.80 ± 0.23	0.73 ± 0.19	0.29	0.005	<0.01	0.110	0.57	0.01
	100-150 ms	0.61 ± 0.18	0.59 ± 0.17	0.60 ± 0.16	0.56 ± 0.13	0.40	0.003	<0.01	0.037	0.71	0.01
	150-200 ms	0.37 ± 0.15	0.35 ± 0.12	0.37 ± 0.13	0.35 ± 0.11	0.98	<0.001	0.02	0.025	0.77	<0.01
	max RFD, ms	0.96 ± 0.27	0.86 ± 0.26	0.93 ± 0.23	0.83 ± 0.21	0.43	0.003	<0.01	0.204	0.81	<0.01
Time RFD max, ms		75.6 ± 20.9	77.5 ± 22.8	82.0 ± 27.2	78.4 ± 22.7	0.30	0.005	0.80	<0.001	0.26	0.01

BFlh, biceps femoris long head; MVIC, maximal voluntary isometric contraction; max RFD, maximal rate of force development; η_p_^2^, partial eta square; *P*, *P* value; RFD, rate of force development; ST, semitendinosus; Time RFD max, time when occurred the maximal rate of force development.

## Discussion

This study aimed to better understand the effects of repeated sprinting on hamstring functional and mechanical properties in both healthy nonathletes and soccer players with and without previous hamstring injury, as sprinting has been reported as the main mechanism of hamstring injuries. In both conducted experiments, repeated sprinting induced knee flexors fatigue by decreasing the MVIC and RFD. In experiment I, the repeated sprinting led to a decrease in passive stiffness of both ST and BFlh, and a slight increase in BFlh active stiffness. However, muscle stiffness responses to repeated sprinting were not consistent between days, suggesting that nonathletes may have a variable stiffness response strategy to sprint-induced fatigue. In experiment II, similar repeated sprinting performance was observed between soccer players with and without previous hamstring injury. In addition, although only the ST passive stiffness decreased after the repeated sprinting, the muscles stiffness and knee flexor maximal and rapid strength responses to sprinting did not differ between limbs with and without previous injury.

### Experiment I

While previous research has clearly demonstrated that sprint velocity decreases along the repeated running sprinting trials,^2,12^ few studies have investigated how repeated sprinting affects the knee flexors force production.^8,12^ To the best of our knowledge, previous research has only reported that knee flexor maximal dynamic strength decreases after repeated running sprinting^8,12^; however, the effects of repeated sprinting on knee flexor RFD has not been investigated. It is known that hamstring force production plays an important role during the propulsion phase of running^20^; thus, the ability of the hamstring to produce rapid force is assumed to be of high importance during sprinting. Consequently, in the 2 replicated sessions of the present study, we observed that knee flexor RFD performance decreased in the early stages of contraction (ie, 0-100 ms) and in the initial stage of late RFD (ie, 100-150 ms; but not 150-200 ms), as well as the maximum RFD (ie, which often occurs during the early RFD interval). A previous review indicated that both early and maximum RFD have a greater magnitude of responses to a fatigue state compared with MVIC.^10^ Such a statement is in accordance with findings observed with the nonathletes denoted by normalized average changes, but also revealed by the greater partial eta square found for knee flexors early (η_p_^2^ = 0.685-0.741) and maximum (η_p_^2^ = 0.691) RFD compared with MVIC (η_p_^2^ = 0.441). However, surprisingly, such a response was not observed with soccer players in experiment II, as a greater magnitude of response was observed for MVIC (η_p_^2^ = 0.307) than early (η_p_^2^ = 0.024-0.110) and maximum (η_p_^2^ = 0.204) RFD. Interestingly, a previous study also reported that amateur soccer players did not change RFD after an effort similar to that of a football match, even in the presence of a considerable decrease in MVIC.^36^ This might be attributed to the greater knee flexors strength endurance of this population, due to the fact that they have greater exposure to repeated sprints compared with nonathletes. This aspect is worthy of further investigation.

To our knowledge, this is the first study to report the effects of repeated sprinting on hamstring muscle passive and active stiffness, through the use of ultrasound-based shear wave elastography. Previous studies using shear wave elastography have interpreted the decrease in passive and active stiffness after fatiguing tasks as a indicators of localized muscle fatigue,^7,24,28,34^ whereas an increase in muscle active stiffness (ie, for a given joint torque production) is an indicator of muscle overloading.^3,24^ However, the results found in the present study were somehow intriguing. For instance, while a reduction of passive stiffness in both ST and BFlh was found, a small increase in BFlh active stiffness was noted. Note, however, that the change in BFlh active stiffness was small, and lower than the interday methodological error; so whether this can be interpreted as a random result is questionable. Nevertheless, the increase in BFlh active stiffness might mean that the fatigue induced by the repeated sprinting may have induced a higher BFlh neural overdemand to execute submaximal knee flexor contraction to compensate for the decrease in passive stiffness, but possibly also to generate a compensation mechanism in relation to the load responses of other agonist muscles. Although this possibility cannot be confirmed as only 2 hamstring muscles were measured, it can be said that the potential BFlh compensation is not due to alterations in ST loading. Note that repeated sprints have been reported to decrease knee flexor strength capacity with greater magnitude compared with hip extensors, and that the production of horizontal force during repeated sprints is largely explained by the decrease in hip extensor strength capacity.^12^ Based on the findings of previous studies on single-joint (ie, knee) fatiguing tasks,^15,24^ repeated sprints would be expected to induce a decrease in ST active stiffness, which was not observed. This fact may have occurred due to many possibilities. One possibility could be due to the redundant number of kinematic strategies that athletes may have adopted during sprints to reduce the ST load. On the other hand, it may be related to the nature of the applied sprinting task (ie, 30 m), which involves a greater component of acceleration and hip extension force production dependence, thus inducing specific hip extension fatigue.^35^ Future research is wanted to further explore this topic.

Moreover, another important finding of this study is that muscle stiffness responses to repeated sprinting were not consistent between testing sessions, which suggests that intra-athlete muscle stiffness responses are highly variable between similar sprinting-induced fatigue tasks. This finding is in accordance with the suggestions of previous research,^1,4^ and reinforces the premise that, due to the high number of body postures that participants can adopt during sprints, the load responses between hamstring heads may be highly variable. Future research may want to explore whether hamstring responses after repeated sprinting are related to the kinematic postures adopted during sprinting.

### Experiment II

Since sprinting is a key aspect of the football game,^14^ but also the most common mechanism of hamstring injuries,^9,11^ knee flexor strength capacity and the mechanical properties of hamstrings in response to repeated sprinting were also examined in soccer players. In accordance with previous research,^16,19^ soccer players reporting previous hamstring injury were slightly older and had higher body mass index. Concerning repeated sprinting performance, soccer players with previous hamstring injury did not differ from noninjured athletes as they had similar maximal sprint velocity and drop in velocity along the repeated sprint task. This result opposes the findings of previous research reporting that soccer players with previous hamstring injury are faster,^22,31^ and have a higher drop in velocity along the repeated sprints compared with noninjured soccer players.^31^ Note, however, that soccer players participating in the present study belonged to third (23%) and fourth (57%) division teams; thus, their competitive level (as well sprint performance level) might be lower than soccer players who participated in the study by Røksund et al.^31^ Also, the maximal sprint velocity was similar between soccer players (experiment II) and nonathletes (experiment I). Thus, the repeated sprinting performance difference between soccer players with versus without previous hamstring injury might be detected only at higher performance/competitive levels.

Similar to the findings of experiment I, the repeated sprinting was also shown to induce knee flexor fatigue, by decreasing MVIC and the RFD (in all intervals). However, the magnitude of the responses was generally lower in athletes compared with nonathletes, as denoted by the partial eta square values (ie, 0.025-0.307 vs 0.089-0.741). On the other hand, muscle stiffness responses after repeated sprinting did not alter as observed in experiment I, with the exception of ST passive stiffness, which exhibited a reduction. We are unaware of the reason for this discordance of results between experiments; however, it may be explained by the degree of fatigue induced by repeated sprints. As mentioned, the magnitude of knee flexor fatigue was higher in soccer players. Also, note that soccer players decreased the sprint velocity only from the fourth trial compared with the first sprint trial, while nonathletes demonstrated a decrease in velocity immediately on the second trial. Perhaps, due to greater exposure to repeated sprints of soccer players (compared with nonathletes), they have developed greater hamstring strength endurance. Consequently, we may speculate that hamstring stiffness changes are evident only with higher levels of fatigue, and 10 repeated sprints with 30 seconds rest between attempts may not be enough to induce such stiffness alterations. Therefore, it is desirable that future research apply protocols that induce greater degrees of fatigue, to verify whether the stiffness of hamstring muscles in football players remains unaltered.

With respect to the differences between limbs with versus without previous hamstring injury, surprisingly, we found no difference for any knee flexor strength or hamstring stiffness variable. Regarding knee flexor RFD, the present results opposes somewhat the findings of Opar et al^30^ and Buhmann et al,^5^ who assessed knee flexor RFD under dynamic conditions (ie, eccentric contractions), but agree with the results of Nara et al,^29^ who assessed knee flexor RFD under isometric conditions. The findings for knee flexor MVIC also agree with those from previous studies,^29^ whereas no differences were found between athletes with versus without previous hamstring injury. Thus, it is possible that knee flexor strength differences between previously injured and noninjured hamstring athletes can be found only in a dynamic contraction regime, in particular in eccentric contractions. Regarding hamstring muscle stiffness, no differences were observed between groups for both fatigue and nonfatigue states; thus, the initial hypotheses of this study was not supported. To the best of our knowledge, using ultrasound shear wave elastography, only 1 previous study has assessed the active stiffness of ST and BFlh during a knee flexor fatiguing task with elite soccer players^15^ and reported that BFlh active stiffness in a nonfatigue state was lower in previously injured limbs. Although, in the present study, the mean BFlh active stiffness values of previously injured limbs were lower compared with those of noninjured limbs, such differences did not reach statistical significance (*P* = 0.13). We speculate that the reason for this discrepancy might be related to the competitive level of soccer players between studies, and the method of injury diagnosis (ie, in the study of Freitas et al,^15^ all the hamstring injuries were diagnosed through MRI, while in the present study they were based on athlete self-report). In terms of passive stiffness, our results are in accordance with those of Kawai et al,^23^ who did not find stiffness differences between injured and noninjured limbs in athletes who suffered injury >6 weeks before testing. Therefore, in nonsevere situations, it is possible that passive stiffness of injured muscles is restored in <6 weeks.

### Study Limitations

There are 3 main study limitations. First, hamstring injury screening was based on athlete self-report, instead of clinical imaging (ie, MRI). This fact did not allow access to the exact muscle injury location and classification, but allowed access to the body region (ie, hamstring), time of injury occurrence, and injury time loss (ie, severity).^17^ Second, active stiffness assessment was performed only at 20% of MVIC. However, it should be noted that the changes in the vastus lateralis active stiffness with fatigue tend to occur over a wide band of contraction intensities^28^; thus, it is unknown whether this is the case for the hamstring muscles. Finally, active stiffness analysis (between participants and limbs) was performed for a relative contraction intensity (ie, percentage of MVIC), instead of an absolute torque. The stiffness comparison for a given knee flexion torque is a possible methodological approach; however, it does not prove that the individual muscle force between participants/limbs is the same. It is necessary to consider other factors, such as muscle moment arm, physiological cross-sectional area, and degree of muscle activation.^21^ However, no differences were found in the knee flexors MVIC (or body mass) between footballers with and without a previous injury, which gives some degree of confidence for the approach taken.

### Conclusion

Repeated sprints are likely to induce fatigue in the knee flexors by decreasing their MVIC and RFD in both athlete and nonathlete populations. However, the magnitude of this effect appears to be smaller in those with greater exposure to repeated sprints, namely football (ie, soccer) players. In nonathletes, repeated sprinting seems to alter hamstring stiffness by decreasing both ST and BFlh passive stiffness, and slightly increasing the BFlh active stiffness. In soccer players, only a passive decrease in ST stiffness was noted. However, in nonathletes, the stiffness responses to repeated sprinting appear to not be consistent between days, suggesting high intraperson variability. Soccer players with previous hamstring injury in the last 2 years appear to have similar repeated sprinting performance compared with noninjured athletes; repeated sprints also appear not to induce different responses in knee flexor strength and hamstring stiffness between lower limbs with and without a history of hamstring injury.
